# Imetelstat Induces Leukemia Stem Cell Death in Pediatric Acute Myeloid Leukemia Patient-Derived Xenografts

**DOI:** 10.3390/jcm11071923

**Published:** 2022-03-30

**Authors:** Sonali P. Barwe, Fei Huang, Edward Anders Kolb, Anilkumar Gopalakrishnapillai

**Affiliations:** 1 Nemours Centers for Childhood Cancer Research and Cancer & Blood Disorders, Nemours Children’s Health, Wilmington, DE 19803, USA; sbarwe@nemours.org (S.P.B.); eakolb@nemours.org (E.A.K.); 2 Geron Corporation, Parsippany, NJ 07054, USA; fhuang@geron.com

**Keywords:** pediatric acute myeloid leukemia, patient-derived xenograft models, imetelstat, telomerase, leukemia stem cells

## Abstract

**Simple Summary:**

About 20% of children with acute myeloid leukemia (AML) experience refractory disease or relapse, despite receiving intensive therapy. Leukemia stem cells (LSC) have the ability to evade chemotherapy and propagate the disease leading to chemoresistance and relapse. Therefore, treatment options that are able to eliminate LSCs are likely to be more effective in prolonging disease-free survival. We have tested the effect of imetelstat, a potent inhibitor of telomerase activity that specifically kills LSCs, on pediatric AML cells in culture and in mouse models. Imetelstat was effective in specifically killing LSCs and extended animal survival when used as a single agent or in combination with chemotherapy or epigenetic drug azacitidine.

**Abstract:**

Acute myeloid leukemia (AML) in children remains deadly, despite the use of maximally intensive therapy. Because leukemia stem cells (LSCs) significantly contribute to chemoresistance and relapse, therapies that specifically target the LSCs are likely to be more beneficial in improving outcome. LSCs are known to have high telomerase activity and telomerase activity is negatively correlated with survival in pediatric AML. We evaluated the preclinical efficacy of imetelstat, an oligonucleotide inhibitor of telomerase activity in patient-derived xenograft (PDX) lines of pediatric AML. Imetelstat treatment significantly increased apoptosis/death of the LSC population in a dose-dependent manner in six pediatric AML PDX lines ex vivo, while it had limited activity on the stem cell population in normal bone marrow specimens. These results were validated in vivo in two distinct PDX models wherein imetelstat as single agent or in combination with chemotherapy greatly reduced the LSC percentage and prolonged median survival. Imetelstat combination with DNA hypomethylating agent azacitidine was also beneficial in extending survival. Secondary transplantation experiments showed delayed engraftment and improved survival of mice receiving imetelstat-treated cells, confirming the diminished LSC population. Thus, our data suggest that imetelstat represents an effective therapeutic strategy for pediatric AML.

## 1. Introduction

Acute myeloid leukemia (AML) is a heterogenous disease characterized by the accumulation of immature blasts owing to a block in the differentiation of progenitor cells of the myeloid origin. One in five pediatric AML patients experience recurrent disease, despite the use of maximally intensive therapy. These treatment options result in significant short-term and long-term toxicities. To drive the survival rate in pediatric AML upward, novel therapies that are targeted specifically towards malignant cells are needed. Leukemia stem cells (LSCs), owing to their self-renewal capacity, not only drive tumor progression but are also the major cause of relapse following chemotherapy [1]. Therefore, eliminating LSCs is essential to prevent relapse and improve outcome. Telomerase activity was found to be essential for LSC function and inhibition of telomerase activity specifically targeted LSCs in mouse models of adult AML [2].

Genome-wide analyses of pediatric AML patients showed notable differences in the mutational landscape compared to adult AML patients [3]. Loss-of-function mutations in *TERT* gene which codes for the reverse transcriptase enzyme, a component of the telomerase complex, are prevalent in adult AML patients [4]. Because of the germ-line origin of these mutations, they are considered risk factors for adult AML. The infrequent mutations in *TERT* gene in pediatric AML did not result in loss of telomerase activity, and are not considered risk factor [5], highlighting the distinction between pediatric AML and adult AML patients. The 5-year survival rate for pediatric AML patients with lower telomerase activity was about 2-fold higher compared to those patients with higher telomerase activity, suggesting telomerase activity could be an important prognostic factor in pediatric AML patients and targeting telomerase activity is likely to be effective in reducing leukemia burden [6]. Therefore, it is necessary to experimentally evaluate the anti-leukemic effect of telomerase inhibition on pediatric AML.

Imetelstat is a 13-mer oligonucleotide that specifically binds to the RNA template of telomerase with high affinity and is a potent, competitive inhibitor of telomerase enzymatic activity [7,8]. Imetelstat has demonstrated clinical activity in patients with hematological malignancies such as myelodysplastic/myeloproliferative neoplasms (MDS/MPN) [9,10,11,12]. Preclinical studies showed anti-LSC activity of imetelstat in MPN and adult AML models in vivo [2,13,14] and pediatric B-cell acute lymphoblastic leukemia in vitro [15]. Further studies investigated the mechanisms underlying imetelstat’s beneficial effects in MPN patients and how imetelstat leads to thrombocytopenia. These studies revealed that anti-neoplastic effects of imetelstat were reliant on inhibition of telomerase activity [16,17]. Robust preclinical evaluation is essential to best translate this new agent successfully into clinical treatment algorithm. In this study, we evaluated the anti-leukemic effect of imetelstat in pediatric AML patient-derived xenograft (PDX) models. Our data suggest that imetelstat could be used as a therapeutic for pediatric AML.

## 2. Materials and Methods

### 2.1. Reagents and Drugs

Imetelstat sodium and mismatch control oligo were provided by Geron Corporation, Foster City, CA, USA. BV785-human CD45, APC-human CD34, Pacific blue-human CD38, FITC conjugated annexin V and propidium iodide were purchased from BioLegend, San Diego, CA, USA.

### 2.2. Pediatric Samples

We have developed and characterized disseminated PDX models of pediatric AML with varying cytogenetic profiles using AML cells from patients treated at the Nemours Children’s Hospital [18,19]. The mouse passaged pediatric AML PDX lines used in this study (*n* = 6) were generated using primary patient samples. Patient characteristics and demographics of patients aged 1–14 years are provided in Table 1. The normal bone marrow samples (*n* = 4) were collected under IRB approved protocol #1345366 and consisted of diagnostic bone marrow specimens that were later identified to not have leukemia (Table 1).

### 2.3. Apoptosis Assay

1 × 10^5^ mouse passaged pediatric AML PDX lines with >98% human CD45+ cells, or normal bone marrow samples were seeded in culture media (SFEMII obtained from StemCell Technologies, Cambridge, MA, USA, supplemented with 50 ng/mL SCF, 25 ng/mL FLT3, 10 ng/mL IL-3, 10 ng/mL IL-6 and 25 ng/mL TPO) per well in a 96-well plate. Imetelstat or mismatch control oligo were diluted in media at a concentration of 2× and added to the cells to make the final concentrations 1 µM, 2.5 µM, 5 µM, 10 µM, or 20 µM. The untreated cells were used as control. The cells were incubated for 96 h at 37 °C, 5% CO_2_ before staining with BV785-human CD45, APC-human CD34, Pacific blue-human CD38, FITC conjugated annexin V and propidium iodide (PI). Stained cells were analyzed by flow cytometry to detect cells in the early stage of apoptosis (Annexin V+/PI−), later stage of cell death (Annexin V+/PI+) and surviving cells (Annexin V−/PI−) in human CD45+ cells and LSC population (CD34+/CD38low).

### 2.4. Analysis of In Vivo Efficacy and Self-Renewal Capacity

NSG-SGM3 mice were injected intravenously with 3 × 10^6^ cells each of either NTPL-377 or DF-2 pediatric AML PDX line. Disease progression was monitored by evaluating the percentage of human cells in mouse peripheral blood at periodic intervals by flow cytometry using fluorescent tagged antibodies against human and mouse CD45. At 2–3 weeks post transplantation, when % engraftment was greater than 0.5%, mice were randomly assigned to following control or treatment groups—(1) Untreated, (2) Imetelstat, (3) mismatch (control oligo), (4) chemotherapy followed by imetelstat, (5) chemotherapy, (6) concurrent treatment of imetelstat and azacitidine, (7) azacitidine. Mice were injected intraperitoneally with 15 mg/kg imetelstat or mismatch control oligo three times a week for 5 weeks. This dosing regimen was chosen based on previous reports showing efficacy with limited toxicity in adult AML and MPN models [2,14]. Standard chemotherapy included cytarabine (50 mg/kg, Qdx5, i.p.) with daunorubicin (1.5 mg/Kg, Qdx3, i.v.) as described previously [20]. Azacitidine was administered (2.5 mg/Kg, Qdx14, i.p.) in indicated mice. The mice were monitored daily and euthanized when any of the predetermined experimental endpoints such as reduced mobility, weight loss greater than 20% body weight, hind limb paralysis, or hunched back, were reached. Kaplan-Meier survival estimates were plotted. Following euthanasia, bone marrows were flushed and evaluated for the presence of human CD45+ cells and LSC population (CD34+/CD38low).

Cells were collected by flushing the bone marrow of mice treated with or without imetelstat or mismatch oligo post euthanasia. 1 × 10^6^ of these cells were injected per each recipient mouse for evaluation of self-renewal capacity by secondary transplantation. Kaplan-Meier survival curves were plotted as described above. Growth curves were plotted based on the periodic evaluation of CD45+ population in mouse peripheral blood.

### 2.5. Statistical Analysis

All the results are expressed as means ± standard deviation (SD). All the single-parameter measurement comparisons were determined using the Student’s *T*-test (GraphPad Prism 9 software, GraphPad, San Diego, CA, USA). All tests were two-sided; *p* < 0.05 was considered statistically significant. For group comparisons, two-way ANOVA was used. For Kaplan-Meier survival curves, *p*-values were calculated using the Log-rank (Mantel-Cox) test.

## 3. Results

### 3.1. Imetelstat Suppresses the Viability of Pediatric AML LSCs in PDX Samples Ex Vivo

**Figure 1:**
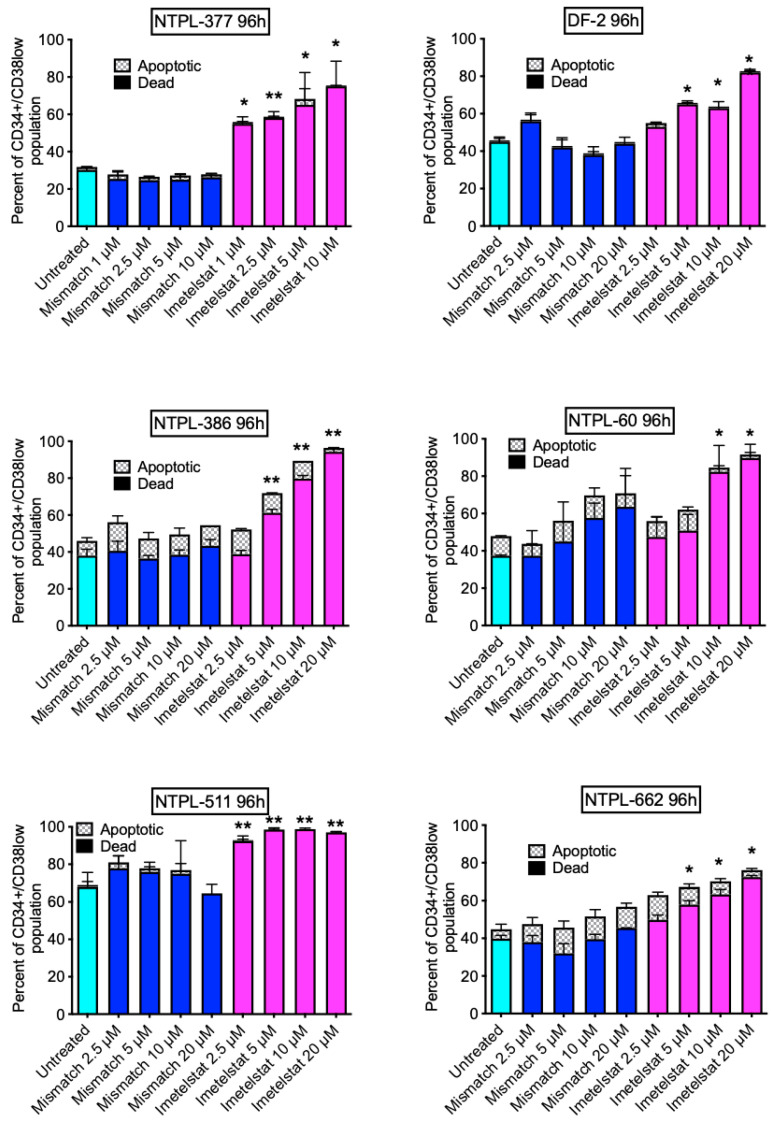
Imetelstat increased the percentage of dead cells in the LSC population in a dose-dependent manner in six pediatric AML PDX lines developed from diagnostic specimens (except DF-2, which was generated from a sample procured at relapse). Error bars denote SE of the mean from 2 independent experiments in triplicates. * *p* < 0.05, ** *p* < 0.01 comparing imetelstat treated cells to mismatch-treated cells at corresponding concentrations.

Mouse passaged pediatric AML PDX lines (Table 1, *n* = 6) were treated ex vivo with either imetelstat or mismatch oligo control. Viability of the LSC population (CD34+CD38low) as well as viability of the total CD45+ human cells was determined at 96 h post treatment by flow cytometry. Gating strategy to calculate the apoptotic and dead cell percentages within the LSC population is shown (Figure S1). Treatment with imetelstat significantly increased the percentage of dead (PI+/annexin V+) cells within the LSC population in a dose-dependent manner in all 6 PDX lines (Figure 1). However, there were no significant changes in the percentage of total human dead cells (Figure S2).

In contrast, imetelstat treatment in general did not show significant impact on the survival of normal stem cells from pediatric bone marrow samples of healthy subjects, only one out of four samples had an increase in PI+/annexin V+ cells in CD34+CD38low cell population with 2.5 µM and 5 µM imetelstat treatment (Figure S3).

### 3.2. Imetelstat Reduces the LSC Population In Vivo and Prolongs Survival in Two PDX Models

**Figure 2:**
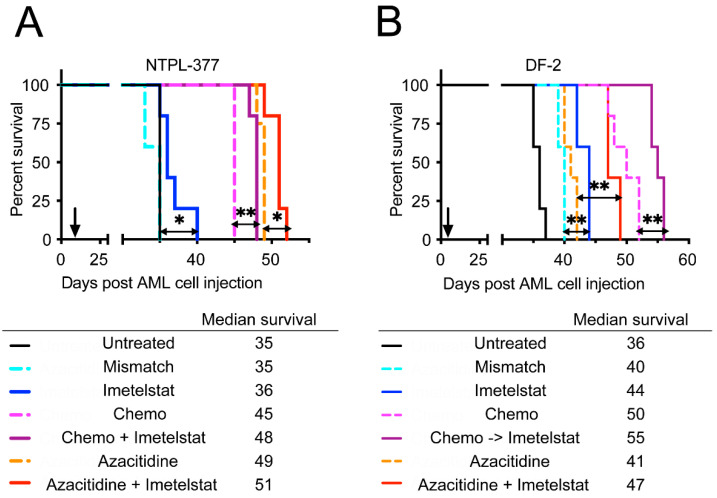
Kaplan-Meier survival plots showing percent survival in NTPL-377 (**A**) and DF-2 (**B**) engrafted mice receiving indicated treatments (*n* = 5 per group). Chemo = chemotherapy, Chemo -> Imetelstat indicates chemotherapy followed by imetelstat. Arrows indicate time when treatment began. * *p* < 0.05, ** *p* < 0.01.

The efficacy of imetelstat as single agent or in combination with chemotherapy (Chemo) was evaluated in two distinct PDX models of pediatric AML. In the NTPL-377 engrafted mice, 4 of 5 mice treated with imetelstat lived longer than the untreated mice or mice treated with mismatch control (Figure 2A, *n* = 5 per group, *p* = 0.0116). Mice receiving standard chemotherapy consisting of cytarabine and daunorubicin showed a 10-day prolonged survival compared to untreated mice. Interestingly, sequential administration of imetelstat following chemotherapy treatment provided additional benefit over chemotherapy (Figure 2A, *p* = 0.0027). In a second PDX model of pediatric AML, DF-2, imetelstat-treated mice lived significantly longer than untreated, or mismatch oligo treated mice (Figure 2B, *n* = 5 per group, *p* = 0.0031). Chemotherapy treated mice exhibited a 14-day increase in median survival compared to untreated mice. Mice with administration of chemotherapy followed by imetelstat survived 5 days longer than chemotherapy only (Figure 2B, *n* = 5 per group, *p* = 0.0027).

We also evaluated the effect of combination of imetelstat with azacitidine, a DNA hypomethylating agent with known anti-leukemic effects. In NTPL-377, azacitidine treatment alone prolonged survival by 14 days; this effect was greater than that of chemotherapy. Concurrent treatment of azacitidine and imetelstat further extended survival of these mice (Figure 2A, *n* = 5 per group, *p* = 0.021). Unlike NTPL-377 model, the improvement in survival by azacitidine treatment (5 days) was smaller than chemotherapy treatment in DF-2. Concurrent treatment with azacitidine and imetelstat prolonged survival by 6 more days compared to azacitidine alone (Figure 2B, *n* = 5 per group, *p* = 0.0025). Taken together, imetelstat treatment prolonged survival either alone or in combination with chemotherapy or azacitidine.

**Figure 3:**
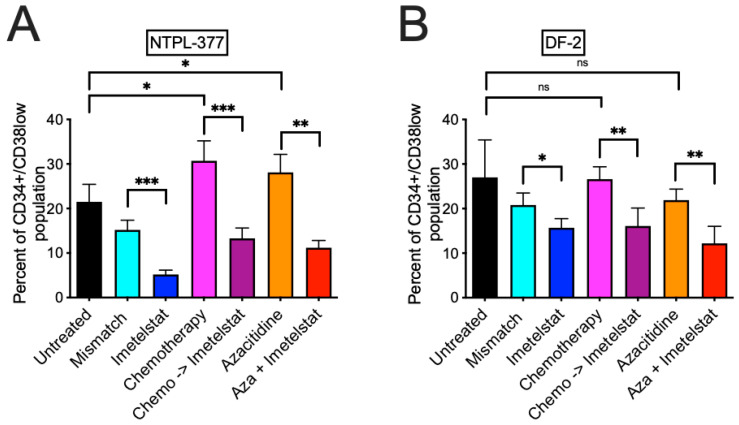
Percentage of LSC population in terminal bone marrow samples isolated from mice post euthanasia in NTPL-377 (**A**) and DF-2 (**B**). Chemo = chemotherapy, Chemo -> Imetelstat indicates chemotherapy followed by imetelstat. * *p* < 0.05, ** *p* < 0.01, *** *p* < 0.001, ns = not significant.

We also estimated the percentage of LSC population (CD34+CD38low population) in the terminal bone marrow samples isolated from mice post euthanasia and compared between groups using two-way ANOVA (Table S1). The percentage of LSC population in mice treated with imetelstat was significantly reduced compared to those treated with the mismatch oligo in both NTPL-377 (Figure 3A, *p* = 0.0002 and Figure S4) and DF-2 (Figure 3B, *p* = 0.0110) models. In contrast with imetelstat, both chemotherapy and azacitidine treatment significantly increased the LSC population in NTPL-377 model (Figure 3A, *p* = 0.0182 and *p* = 0.0489, respectively); neither chemotherapy alone nor azacitidine as a single agent significantly altered the LSC population in comparison with untreated mice in DF-2 model. A significant reduction in the LSC population was observed when imetelstat was combined with chemotherapy or azacitidine compared to chemotherapy or azacitidine single agent in NTPL-377 (Figure 3A, *p* = 0.0009 and *p* = 0.0011, respectively, Figure S4) and DF-2 (Figure 3B, *p* = 0.0091 and *p* = 0.0008, respectively).

### 3.3. Imetelstat Delays Engraftment and Improves Survival in Secondary Transplantation Model

**Figure 4:**
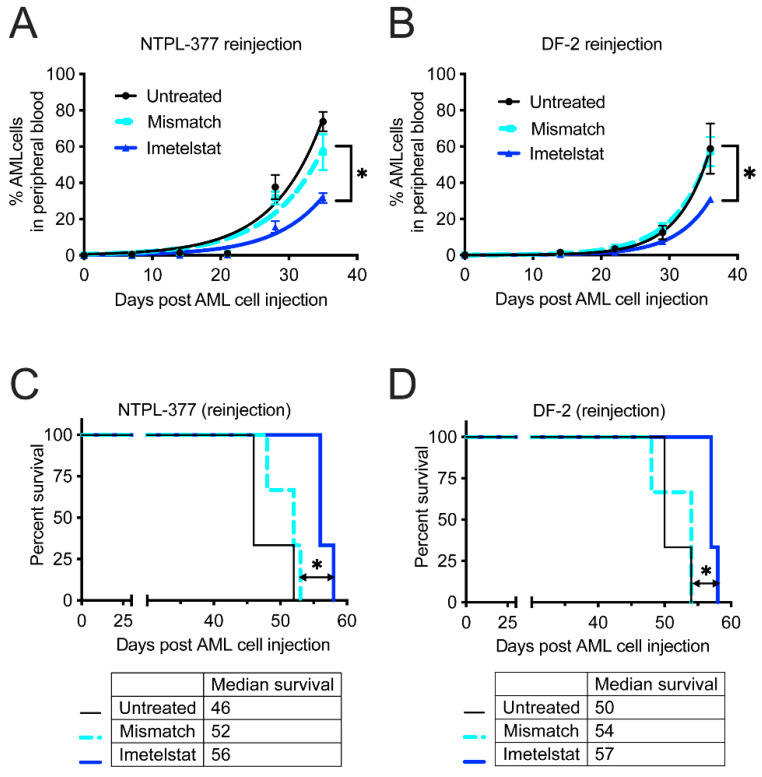
Imetelstat treatment delays engraftment and improves mouse survival in a secondary transplantation study. (**A**,**B**) Growth curves showing the rise in the percentage of AML (CD45+) cells in mouse peripheral blood. * *p* < 0.05. (**C**,**D**) Kaplan-Meier survival plots showing the percent survival in mice, *n* = 3 each.

The reduction in LSC population in vivo following imetelstat treatment was further confirmed by secondary transplantation in mice. This experiment showed delayed engraftment of cells isolated from imetelstat treated mice compared to untreated or mismatch oligo treated mice in both PDX models (Figure 4A, *p* = 0.0129, and Figure 4B, *p* = 0.0330, *n* = 3 per group each). Furthermore, there was a significant improvement in the survival of mice injected with cells isolated from imetelstat treated mice in both PDX models (Figure 4C, *p* = 0.0246, and Figure 4D, *p* = 0.0296, *n* = 3 each).

## 4. Discussion

It has been established that the genomic landscape of pediatric AML is distinct from adult AML [3]. Therefore, preclinical evaluation of therapy options is encouraged before clinical use of novel drugs in children. We tested the effect of imetelstat ex vivo using the well-characterized PDX lines derived from pediatric AML patients ranging from 1–14 years of age and covering different FAB subtypes with diverse cytogenetic abnormalities, representing pediatric AML patient population. The efficacy of imetelstat as a single agent was demonstrated in these pediatric AML PDX models, with (1) increased apoptosis/death of LSC population ex vivo (Figure 1) with minimal activity in the stem cells of normal pediatric bone marrow samples (Figure S3); (2) reduction in LSC population in bone marrow of mice (Figure 3), which was associated with prolonged survival (Figure 2) and (3) inhibition of LSC’s self-renewal property which delayed engraftment in a secondary transplant model resulting in improved survival (Figure 4). These results corroborated with data from preclinical studies in adult AML [2], and MPN [13,14] models.

Clinical studies demonstrated that inhibition of telomerase activity and reduction in hTERT by imetelstat treatment correlated with efficacy [10,11]. It was reported that imetelstat treatment reduced telomerase activity leading to apoptosis/cell death in malignant hematopoietic stem cells of adult AML and MPN [2,14]. Because of short treatment duration (96 h), the apoptosis induction observed by imetelstat in the ex vivo study may not be necessarily due to telomere shortening but is likely to be the on-target action of imetelstat by telomerase inhibition. Although we did not measure the telomerase activity post imetelstat treatment in this study, we compared the effectiveness of imetelstat with mismatch oligo in both ex vivo and in vivo studies. The only difference between these two oligos is the ability of imetelstat to bind to the telomerase RNA template with high affinity and specifically inhibit telomerase activity. The mismatch control oligo had ~2000-fold higher IC_50_ for inhibition of telomerase activity, indicating a high level of sequence specificity and minimal non-specific inhibitory activity of imetelstat [7]. Telomerase activity in pediatric AML blasts was higher compared to normal control, and pediatric AML patients with higher telomerase activity had poor survival compared with patients with lower telomerase activity [6]. This suggests that the anti-LSC activity by imetelstat observed in this preclinical study is likely via telomerase inhibition. The limitation of this study was the lack of investigation on mechanism of action. It remains to be determined if suppression of telomerase enzymatic activity and downregulation of hTERT by imetelstat correlates with the observed anti-leukemic effect in pediatric AML.

Chemotherapy drugs daunorubicin and cytarabine are often used to treat pediatric AML patients. Chemoresistance or relapse may occur due to different mechanisms including residual LSCs, which are chemoresistant cells capable of self-renewal and promote the maintenance and propagation of AML. Compared to CD34+/CD38+ blasts, CD34+/CD38- LSCs have reduced in vitro sensitivity to daunorubicin, thereby conferring chemotherapy resistance [21,22,23]. Indeed, chemotherapy treatment of pediatric AML PDX models either had increased or no impact on the LSC population (Figure 3). Imetelstat treatment following chemotherapy significantly enhanced survival of treated mice (Figure 2), probably due to imetelstat’s ability to eradicate the LSC population in bone marrow (Figure 3) while chemotherapy eliminated bulk disease. It is noted that the enhanced survival of mice with imetelstat alone or in combination was limited. Longer and durable effects including reduction in cytogenetically abnormal clones and mutational burden were obtained by continuous administration of imetelstat in MDS/MPN clinical trials [9,10,11,12]. Considering the poor prognosis of AML, novel therapies are needed to target LSCs. Imetelstat may fill this gap by eliminating LSCs while sparing normal stem cells.

Higher methylation status of the promoter/exon1 region of the gene *TERT*, which codes for the reverse transcriptase subunit of the telomerase, in leukemia cell lines showed significant association with reduced specific killing by imetelstat [24]. The DNA hypomethylating agent azacitidine has been shown to inhibit the expression of *TERT* in AML cell lines and primary cells derived from adult AML patients [25]. Combination of imetelstat and azacitidine demonstrated enhanced activity in AML cell lines from adult patients [24,26]. We previously showed that azacitidine, a known anti-leukemia drug, has activity in pediatric AML xenograft models [27]. In this study, we evaluated the imetelstat and azacitidine combination in pediatric AML PDX models with a goal of increased efficacy, considering imetelstat’s effect on LSCs, which are not eradicated by epigenetic drug treatment and are therefore potential causes of resistance and disease relapse. Our study showed that azacitidine itself did not reduce LSC population; rather, it increased LSC percentage in NTPL-377 model, consistent with previous reports on the inability of azacitidine to eradicate LSC population [28]. When imetelstat was combined with azacitidine, a significant reduction in the LSC population was observed (Figure 3). We observed enhanced median survival in mice treated with azacitidine and imetelstat combination, indicating that this may be a rational combination.

## 5. Conclusions

Imetelstat treatment of pediatric AML PDX samples showed significant dose-dependent effects on the viability of the LSCs to induce cell apoptosis/death, while it had limited effect on normal stem cells. This differential activity between samples from pediatric AML patients and normal children in this study supports that imetelstat has the ability to eradicate LSC while sparing normal cells. Our in vivo data were consistent with the ex vivo observation of reduced viability of the LSC population. Imetelstat specifically affects the LSC population was confirmed in two ways—(1) the percentage of LSC population was significantly lower in the bone marrow of imetelstat-treated mice, and (2) secondary transplantation study showed reduced growth rate and slow engraftment in mice treated with imetelstat. In both PDX models, combination of imetelstat with either chemotherapy or azacitidine yielded significantly greater improvement in median survival and reduction in LSC population compared to single agent treatment with either modality. Thus, we believe our data provide the preclinical validation for clinical evaluation of imetelstat in pediatric AML.

## Figures and Tables

**Table 1:** Patient demographics, characteristics, and cytogenetics of pediatric AML PDX lines.

Sample	Ethnicity	Age (Years)/Sex	AML Sub Type	Sample Collected at	FISH	Karyotype	Genomics (Archer Panel)
NTPL-60	African American	4/M	M7	Diagnosis	Trisomy 21, AML1 and ETO amplification	46, XY der (14;21) (q10;q10), +21c [cp12]/48, idem, +8, +der (14;21) (q10; q10) [cp8}−	*GATA1* mutation
NTPL-377	Hispanic	1.5/F	M5	Diagnosis	*KMT2A* rearrangement	46, XX, t(9;11)(p21;q23)[20]	*KMT2A-MLLT3* (56%)
NTPL-386	Non-Hispanic	2/M	M7	Diagnosis	Trisomy 21, RUNX1 amplification	47,XY,del(13)(q12q14),+21c [12]/47,ldem,l(7)(q10)[3]/47,XY,+21c[5]	*GATA1* mutation; *KMT2A-TMEM25* (8%)
NTPL-511	Unknown	14/M	M2	Diagnosis	Negative	47, XY,+8[1]/46,XY[29]	*NUP98-NSD1* (20%); *NSD1-NUP98* (7%)
NTPL-662	Unknown	14/M	M7	Diagnosis	Trisomy 21, low level trisomy 8	47, XY,+21c[91]/48,idem,+8[2]	none
DF-2 (CBAM-68552)	Caucasian	1/M	M5	Relapse following chemotherapy	*KMT2A* rearrangement	46,XY,inv(6)(q23q27)[20]	*KMT2A-MLLT4* (54%)
NTPL-257	Caucasian	3/F		Normal			
NTPL-793	Caucasian	13/F		Normal			
NTPL-827	African American	0.6/M		Normal			
NTPL-837	African American	2/M		Normal			

## Data Availability

Data presented in this article is available in here and in Supplementary Material.

## Supplementary Materials

The following supporting information can be downloaded at: https://www.mdpi.com/article/10.3390/jcm11071923/s1, Figure S1: Gating strategy to evaluate the effect of imetelstat on LSC population in pediatric AML PDX lines and normal CD34+Cd38low cells. Figure S2: Imetelstat had minimal effect on the percentage of dead cells in pediatric AML PDX lines. Figure S3: Effect of imetelstat on the viability of CD34+CD38low population in normal pediatric bone marrow samples at 96 h post treatment. Figure S4: Representative flow cytometry plots showing the LSC (CD34+/CD38low) population. Table S1: Two-way ANOVA analysis to compare the effect between treatment groups on percentage of LSC population (CD34+CD38low population) in the terminal bone marrow samples isolated from mice treated with either single agent or in combination.

## References

[1] Gentles A.J., Plevritis S.K., Majeti R., Alizadeh A.A. (2010). Association of a leukemic stem cell gene expression signature with clinical outcomes in acute myeloid leukemia. JAMA.

[2] Bruedigam C., Bagger F.O., Heidel F.H., Paine Kuhn C., Guignes S., Song A., Austin R., Vu T., Lee E., Riyat S. (2014). Telomerase inhibition effectively targets mouse and human AML stem cells and delays relapse following chemotherapy. Cell Stem Cell.

[3] Bolouri H., Farrar J.E., Triche T., Ries R.E., Lim E.L., Alonzo T.A., Ma Y., Moore R., Mungall A.J., Marra M.A. (2018). The molecular landscape of pediatric acute myeloid leukemia reveals recurrent structural alterations and age-specific mutational interactions. Nat. Med..

[4] Calado R.T., Regal J.A., Hills M., Yewdell W.T., Dalmazzo L.F., Zago M.A., Lansdorp P.M., Hogge D., Chanock S.J., Estey E.H. (2009). Constitutional hypomorphic telomerase mutations in patients with acute myeloid leukemia. Proc. Natl. Acad. Sci. USA.

[5] Aalbers A.M., Calado R.T., Young N.S., Zwaan C.M., Wu C., Kajigaya S., Coenen E.A., Baruchel A., Geleijns K., de Haas V. (2013). Telomere length and telomerase complex mutations in pediatric acute myeloid leukemia. Leukemia.

[6] Verstovsek S., Manshouri T., Smith F.O., Giles F.J., Cortes J., Estey E., Kantarjian H., Keating M., Jeha S., Albitar M. (2003). Telomerase activity is prognostic in pediatric patients with acute myeloid leukemia: Comparison with adult acute myeloid leukemia. Cancer.

[7] Asai A., Oshima Y., Yamamoto Y., Uochi T.A., Kusaka H., Akinaga S., Yamashita Y., Pongracz K., Pruzan R., Wunder E. (2003). A novel telomerase template antagonist (GRN163) as a potential anticancer agent. Cancer Res..

[8] Herbert B.S., Gellert G.C., Hochreiter A., Pongracz K., Wright W.E., Zielinska D., Chin A.C., Harley C.B., Shay J.W., Gryaznov S.M. (2005). Lipid modification of GRN163, an N3’→P5’ thio-phosphoramidate oligonucleotide, enhances the potency of telomerase inhibition. Oncogene.

[9] Baerlocher G.M., Oppliger Leibundgut E., Ottmann O.G., Spitzer G., Odenike O., McDevitt M.A., Roth A., Daskalakis M., Burington B., Stuart M. (2015). Telomerase Inhibitor Imetelstat in Patients with Essential Thrombocythemia. N. Engl. J. Med..

[10] Mascarenhas J., Komrokji R.S., Palandri F., Martino B., Niederwieser D., Reiter A., Scott B.L., Baer M.R., Hoffman R., Odenike O. (2021). Randomized, Single-Blind, Multicenter Phase II Study of Two Doses of Imetelstat in Relapsed or Refractory Myelofibrosis. J. Clin. Oncol..

[11] Steensma D.P., Fenaux P., Van Eygen K., Raza A., Santini V., Germing U., Font P., Diez-Campelo M., Thepot S., Vellenga E. (2021). Imetelstat Achieves Meaningful and Durable Transfusion Independence in High Transfusion-Burden Patients with Lower-Risk Myelodysplastic Syndromes in a Phase II Study. J. Clin. Oncol..

[12] Tefferi A., Lasho T.L., Begna K.H., Patnaik M.M., Zblewski D.L., Finke C.M., Laborde R.R., Wassie E., Schimek L., Hanson C.A. (2015). A Pilot Study of the Telomerase Inhibitor Imetelstat for Myelofibrosis. N. Engl. J. Med..

[13] Ma W., Mason C., Chen P., Jiang Q., Delos Santos N., Lazzari E., Morris S., Mondala P., Isquith J., Huang F. (2017). Telomerase Inhibition Impairs Self-Renewal of ß-Catenin Activated Myeloproliferative Neoplasm Progenitors. Blood.

[14] Wang X., Hu C.S., Petersen B., Qiu J., Ye F., Houldsworth J., Eng K., Huang F., Hoffman R. (2018). Imetelstat, a telomerase inhibitor, is capable of depleting myelofibrosis stem and progenitor cells. Blood Adv..

[15] Karow A., Haubitz M., Oppliger Leibundgut E., Helsen I., Preising N., Steiner D., Dantonello T.M., Ammann R.A., Roessler J., Kartal-Kaess M. (2021). Targeting Telomere Biology in Acute Lymphoblastic Leukemia. Int. J. Mol. Sci..

[16] Mosoyan G., Kraus T., Ye F., Eng K., Crispino J.D., Hoffman R., Iancu-Rubin C. (2017). Imetelstat, a telomerase inhibitor, differentially affects normal and malignant megakaryopoiesis. Leukemia.

[17] Baerlocher G.M., Haubitz M., Braschler T.R., Brunold C., Burington B., Oppliger Leibundgut E., Go N. (2019). Imetelstat inhibits growth of megakaryocyte colony-forming units from patients with essential thrombocythemia. Blood Adv..

[18] Gopalakrishnapillai A., Kolb E.A., Dhanan P., Bojja A.S., Mason R.W., Corao D., Barwe S.P. (2016). Generation of Pediatric Leukemia Xenograft Models in NSG-B2m Mice: Comparison with NOD/SCID Mice. Front. Oncol..

[19] Barwe S.P., Gopalakrisnapillai A., Mahajan N., Druley T.E., Kolb E.A., Crowgey E.L. (2020). Strong concordance between RNA structural and single nucleotide variants identified via next generation sequencing techniques in primary pediatric leukemia and patient-derived xenograft samples. Genom. Inf..

[20] Gopalakrishnapillai A., Correnti C.E., Pilat K., Lin I., Chan M.K., Bandaranayake A.D., Mehlin C., Kisielewski A., Hamill D., Kaeding A.J. (2021). Immunotherapeutic Targeting of Mesothelin Positive Pediatric AML Using Bispecific T Cell Engaging Antibodies. Cancers.

[21] Costello R.T., Mallet F., Gaugler B., Sainty D., Arnoulet C., Gastaut J.A., Olive D. (2000). Human acute myeloid leukemia CD34+/CD38- progenitor cells have decreased sensitivity to chemotherapy and Fas-induced apoptosis, reduced immunogenicity, and impaired dendritic cell transformation capacities. Cancer Res..

[22] van Gils N., Denkers F., Smit L. (2021). Escape From Treatment; the Different Faces of Leukemic Stem Cells and Therapy Resistance in Acute Myeloid Leukemia. Front. Oncol..

[23] Hanekamp D., Cloos J., Schuurhuis G.J. (2017). Leukemic stem cells: Identification and clinical application. Int. J. Hematol..

[24] Zhao X., Tian X., Kajigaya S., Cantilena C.R., Strickland S., Savani B.N., Mohan S., Feng X., Keyvanfar K., Dunavin N. (2016). Epigenetic landscape of the TERT promoter: A potential biomarker for high risk AML/MDS. Br. J. Haematol..

[25] Zhang X., Li B., de Jonge N., Björkholm M., Xu D. (2015). The DNA methylation inhibitor induces telomere dysfunction and apoptosis of leukemia cells that is attenuated by telomerase over-expression. Oncotarget.

[26] Rusbuldt J., Bussolari J., Rizo A., Huang F. (2016). Abstract 2731: Impact of hypomethylating agents on hTERT expression and synergistic effect in combination with imetelstat, a telomerase inhibitor, in AML cell lines. Cancer Res..

[27] Gopalakrishnapillai A., Kolb E.A., McCahan S.M., Barwe S.P. (2017). Epigenetic drug combination induces remission in mouse xenograft models of pediatric acute myeloid leukemia. Leuk Res..

[28] Craddock C., Quek L., Goardon N., Freeman S., Siddique S., Raghavan M., Aztberger A., Schuh A., Grimwade D., Ivey A. (2013). Azacitidine fails to eradicate leukemic stem/progenitor cell populations in patients with acute myeloid leukemia and myelodysplasia. Leukemia.

