# Supplementary material for: Imetelstat Induces Leukemia Stem Cell Death in Pediatric Acute Myeloid Leukemia Patient-Derived Xenografts
Source: J Clin Med. 2022 Mar 30;11(7):1923. doi: 10.3390/jcm11071923 (PMC8999576; doi:10.3390/jcm11071923)
Supplement: Supplementary file 1 [file jcm-11-01923-s001.zip › jcm-1637531-supplementary.pdf]

Imetelstat Induces Leukemia Stem Cell Death in Pediatric Acute Myeloid Leukemia Patient-derived Xenografts

Sonali P. Barwe[1], Fei Huang[2], E. Anders Kolb[1], Anilkumar Gopalakrishnapillai[1] *

[1]Nemours Centers for Childhood Cancer Research and Cancer & Blood Disorders, Nemours Children's Health, Wilmington, DE 19803

[2]Geron Corporation, Parsippany, NJ 07054

*Address of correspondence:

Anilkumar Gopalakrishnapillai

Nemours Center for Childhood Cancer Research

Nemours Children's Health

Wilmington, DE 19803

Phone: (302) 651-4833

Fax: (302) 651-4827

Email: anil.g@nemours.org

Keywords: Pediatric acute myeloid leukemia, patient-derived xenograft models, Imetelstat, telomerase, leukemia stem cells

Disclosures: F.H. is an employee of Geron Corporation. Other authors declare no potential conflicts of interest.

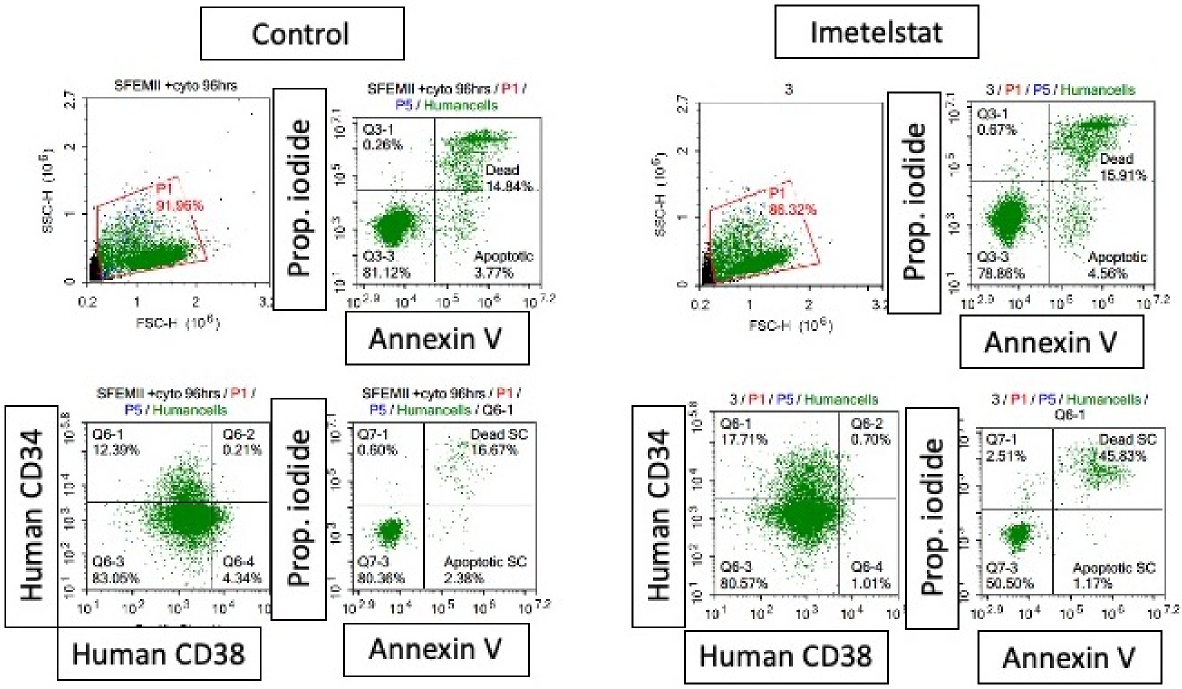

Figure S1 Gating strategy to evaluate the effect of imetelstat on LSC population in pediatric AML PDX lines and normal CD34+Cd38low cells.

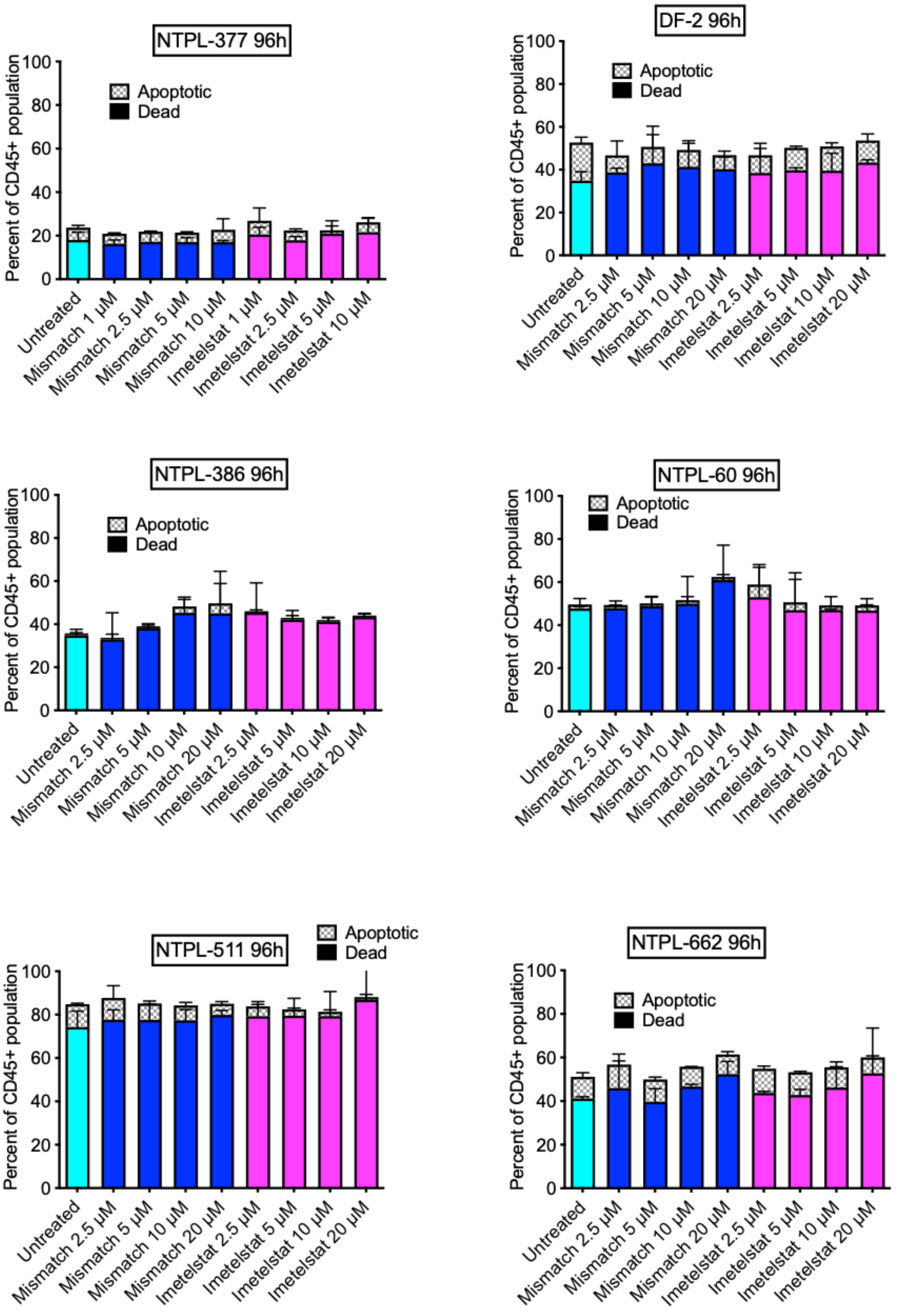

Figure S2 Imetelstat had minimal effect on the percentage of dead cells in pediatric AML PDX lines. Error bars denote SD of the mean from 2 independent experiments in duplicates.

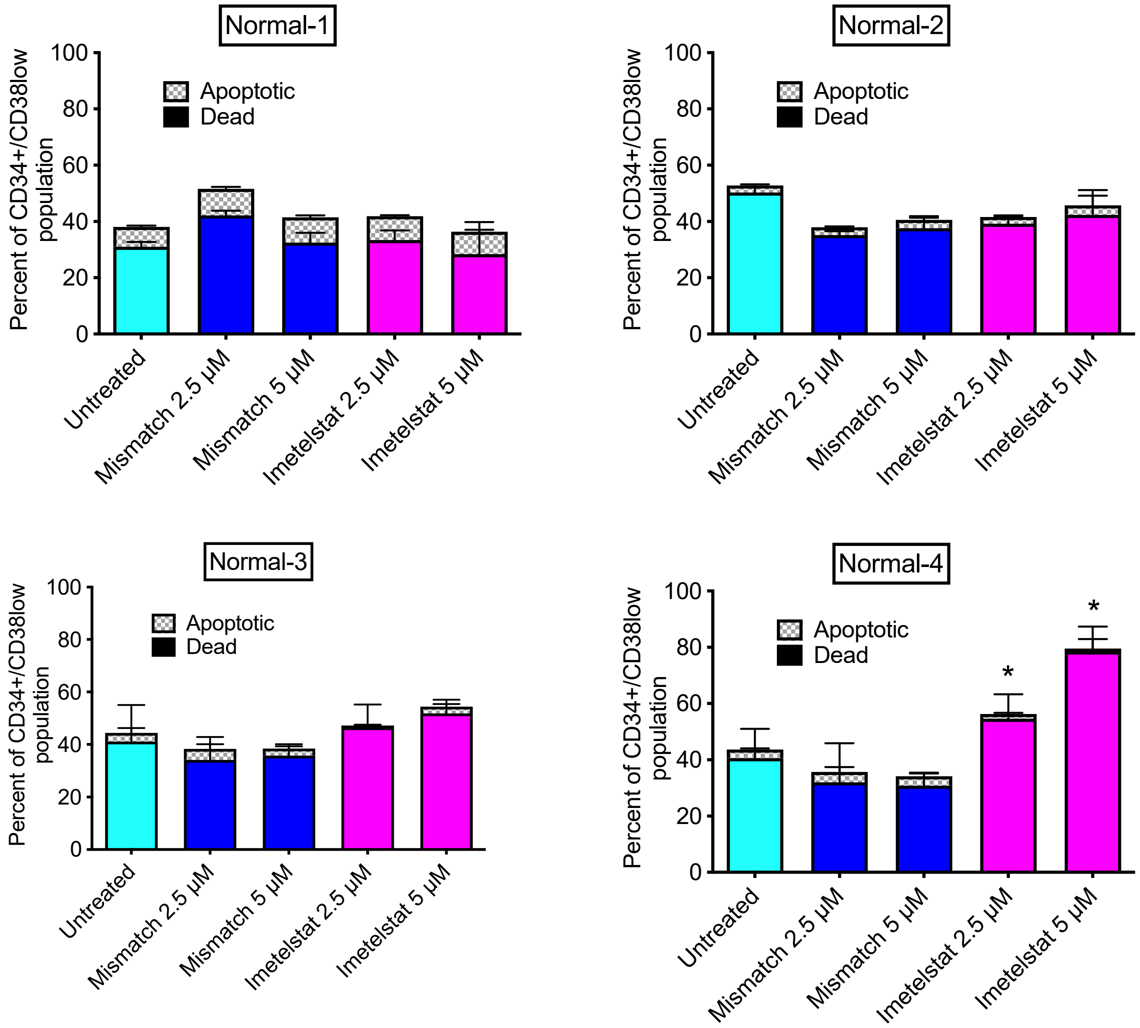

Figure S3 Effect of imetelstat on the viability of CD34+CD38low population in normal pediatric bone marrow samples at 96h post treatment. Error bars denote SD of the mean from 2 independent experiments. *P < 0.05.

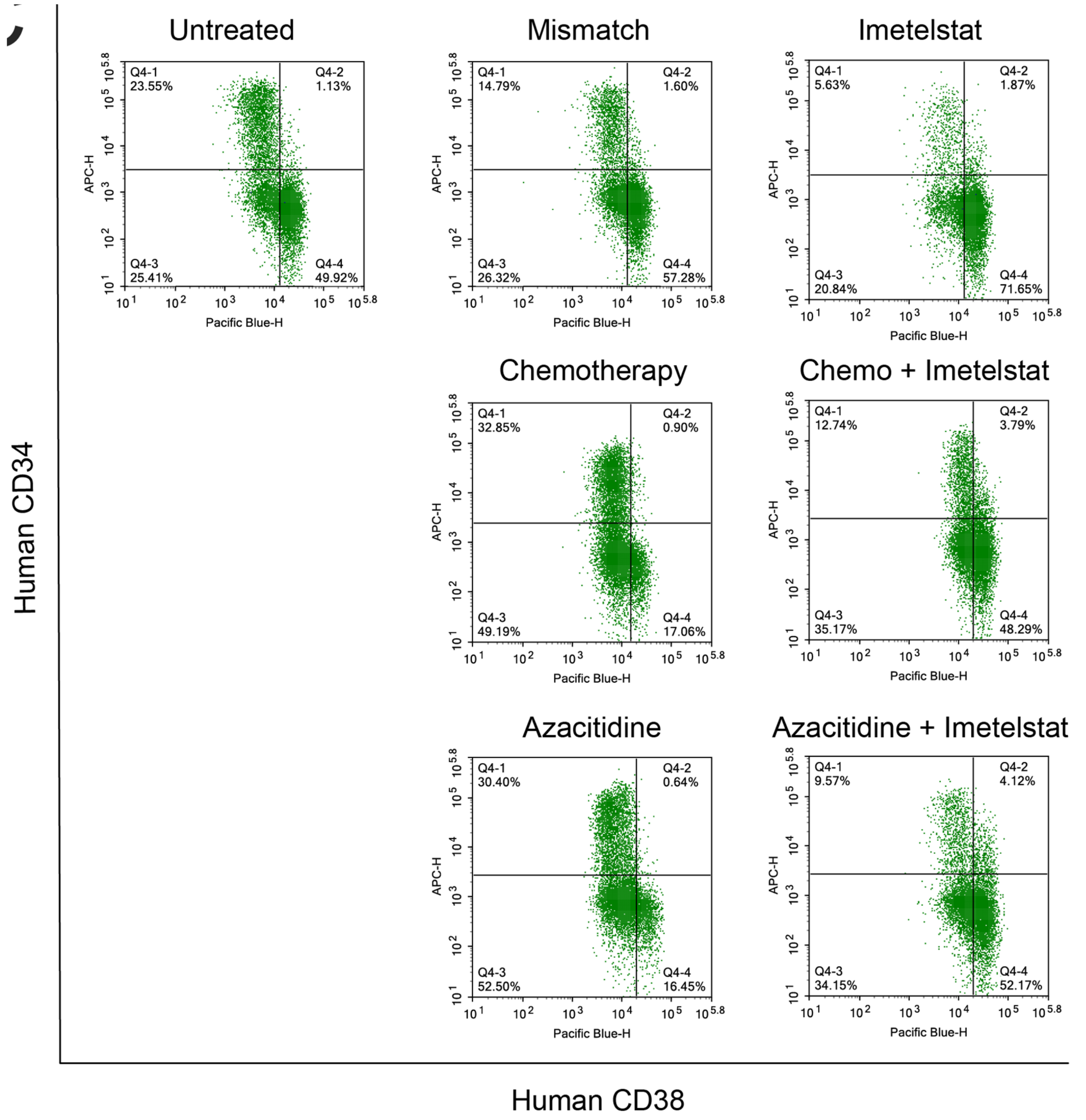

Figure S4 Representative flow cytometry plots showing the LSC (CD34+/CD38^low) population (See upper left quadrant, Q4-1).

Supplementary Table S1
Two-way ANOVA analysis to compare the effect between treatment groups on percentage of LSC population (CD34+CD38low population) in the terminal bone marrow samples isolated from mice treated with either single agent or in combination.

NTPL-377					
ANOVA table	Sums of Squares (SS) Type III	Degrees of freedom (DF)	Mean SS	F (DFn, DFd)	P value
Interaction	84.32	2	42.16	F (2, 19) = 6.680	P=0.0064
Column Factor (Mismatch or **Imetelstat**)	1411	1	1411	F (1, 19) = 223.6	P<0.0001
Row Factor (**Combination**: chemotherapy or azacitidine)	637.7	2	318.8	F (2, 19) = 50.52	P<0.0001

DF-2					
ANOVA table	Sums of Squares (SS) Type III	Degrees of freedom (DF)	Mean SS	F (DFn, DFd)	P value
Interaction	41.44	2	20.72	F (2, 22) = 3.126	P=0.0638
Column Factor (Mismatch or **Imetelstat**)	494.1	1	494.1	F (1, 22) = 74.55	P<0.0001
Row Factor (**Combination**: chemotherapy or azacitidine)	88.07	2	44.04	F (2, 22) = 6.644	P=0.0055
